# Genomic and Phenotypic Insights into the Potential of *Bacillus subtilis* YB-15 Isolated from Rhizosphere to Biocontrol against Crown Rot and Promote Growth of Wheat

**DOI:** 10.3390/biology11050778

**Published:** 2022-05-20

**Authors:** Wen Xu, Qian Yang, Xia Xie, Paul H. Goodwin, Xiaoxu Deng, Jie Zhang, Runhong Sun, Qi Wang, Mingcong Xia, Chao Wu, Lirong Yang

**Affiliations:** 1 Henan International Joint Laboratory of Crop Protection, Henan Biopesticide Engineering Research Center, Institute of Plant Protection Research, Henan Academy of Agricultural Sciences, Zhengzhou 450002, China; xuwen@hnagri.org.cn (W.X.); yangqian@hnagri.org.cn (Q.Y.); xiexia@hnagri.org.cn (X.X.); ddengxiaoxu@hnagri.org.cn (X.D.); zhangjie@hnagri.org.cn (J.Z.); sunrunhong@hnagri.org.cn (R.S.); xiamingcong@hnagri.org.cn (M.X.); wuchao@hnagri.org.cn (C.W.); 2 School of Agricultural Sciences, Zhengzhou University, Zhengzhou 450001, China; 3 School of Environmental Sciences, University of Guelph, 50 Stone Road East, Guelph, ON N1G2W1, Canada; pgoodwin@uoguelph.ca; 4 College of Plant Protection, China Agricultural University, Beijing 100083, China; wangqi@cau.edu.cn

**Keywords:** *Bacillus subtilis*, *Fusarium pseudograminearum*, wheat, biocontrol agent, genome characterization, growth promotion

## Abstract

**Simple Summary:**

Biological control of plant diseases caused by fungal pathogens using antagonistic microorganisms including *Bacilli* has been considered to be an effective and safe alternative to chemical fungicides. *Fusarium* crown rot of wheat is a serious fungal disease affecting yield and grain quality. In this study, a newly isolated strain of *Bacillus subtilis* YB-15 from soil of wheat rhizosphere significantly inhibited *Fusarium* crown rot as well as improved growth of wheat seedlings. Multiple potential biocontrol and growth-promoting attributes of *Bacillus subtilis* YB-15 were determined in vitro and according to the whole genome sequencing analysis. Overall, the results demonstrated that *Bacillus subtilis* YB-15 has great potential for practical application in controlling plant fungal diseases and improving plant growth.

**Abstract:**

*Fusarium* crown rot caused by *Fusarium pseudograminearum* is one of the most devastating diseases of wheat worldwide causing major yield and economic losses. In this study, strain YB-15 was isolated from soil of wheat rhizosphere and classified as *Bacillus subtilis* by average nucleotide identity analysis. It significantly reduced *Fusarium* crown rot with a control efficacy of 81.50% and significantly improved the growth of wheat seedlings by increasing root and shoot fresh weight by 11.4% and 4.2%, respectively. Reduced *Fusarium* crown rot may have been due to direct antagonism by the production of β-1, 3-glucanase, amylase, protease and cellulase, or by the ability of *B. subtilis* YB-15 to induce defense-related enzyme activities of wheat seedlings, both alone and in seedlings infected with *F. pseudograminearum*. Improved plant growth may be related to the ability of *B. subtilis* YB-15 to secrete indole acetic acid and siderophores, as well as to solubilize phosphorus. In addition, the genome of strain YB-15 was determined, resulting in a complete assembled circular genome of 4,233,040 bp with GC content of 43.52% consisting of 4207 protein-encoding genes. Sequencing the *B. subtilis* YB-15 genome further revealed genes for encoding carbohydrate-active enzymes, biosynthesis of various secondary metabolites, nutrient acquisition, phytohormone production, chemotaxis and motility, which could explain the potential of strain YB-15 to be plant growth-promoting bacteria and biological control agent. *B. subtilis* YB-15 appears to be a promising biocontrol agent against *Fusarium* crown rot as well as for wheat growth promotion.

## 1. Introduction

Wheat (*Triticum* *aestivum* L.) is one of the most important food crops in the world [1]. However, in many wheat-producing countries, yields are significantly reduced by *Fusarium* crown rot predominantly caused by *Fusarium graminearum*, *Fusarium pseudograminearum* and *Fusarium culmorum* [2]. However, *F. pseudograminearum* is more common in drier and warmer areas [3]. At present, the application of chemical fungicides is the principal approach to controlling *Fusarium* crown rot, but long-term use of fungicides can lead to negative environmental effects and reduced effectiveness due to fungicide resistance [4]. More sustainable and ecologically friendly alternatives are needed for *Fusarium* crown rot management [5]. Recently, there have been a number of studies on the biocontrol of wheat *Fusarium* crown rot as an alternative strategy [6,7,8,9].

*Bacillus* species, such as *Bacillus velezensis* and *Bacillus subtilis*, have been widely studied for their strong antagonistic activities against many plant pathogens and their abilities to promote growth [10,11,12,13,14]. They can protect crops against a broad range of fungal pathogens by both indirect and direct mechanisms, including secretion of antimicrobial compounds such as antibiotics and hydrolytic enzymes, competition for resources and triggering induced systemic resistance (ISR) [11,12]. Moreover, *Bacillus* species can promote plant growth through improving nutrient availability, reducing the severity of environmental stresses and altering plant growth hormone homeostasis [10,12,15]. There have been several reports of growth promotion of wheat by *Bacillus* species [16,17,18]. There have also been a few reports of *Bacillus* species biologically preventing *F. pseudograminearum* from causing crown rot on wheat and sorghum [19,20].

With the rapid progress of DNA sequencing technology, high-throughput sequencing has been applied to assemble and analyze many bacterial genomes. For example, the genome of *B. subtilis* PMB102 isolated from leaf of tomato has been sequenced, and genes related to growth promotion, including acetoin and siderophore production, and genes related to disease control, such as exopolysaccharide synthesis and cell wall-degrading enzymes, were found [21]. Another example is an analysis of the 13 genomes of *B. subtilis* strains from soil where the production of non-ribosomal peptides was different among the strains, and in vitro antagonism assays showed that plipastatin alone was enough to inhibit *Fusarium* spp., but both surfactin and plipastatin were required to inhibit *Botrytis cinerea* [22]. Sequencing the *B. subtilis* EA-CB0575 genome revealed that it has homologous genes involved in plant growth promotion, such as the release of volatile compounds, solubilizing phosphate, fixing nitrogen, and producing indole and siderophore [23]. Thus, whole-genome sequencing of biological control agents (BCA)s and plant growth-promoting bacteria (PGPB) is a valuable resource for exploring the characteristics responsible for biocontrol and growth promotion.

In this study, strain YB-15 was isolated from rhizospheric soil of wheat and investigated for its ability to inhibit *Fusarium* crown rot caused by *F. pseudograminearum* as well as to improve the growth of wheat seedlings. To understand its effects, five wheat defense-related enzyme activities were examined following inoculation with strain YB-15 alone, *F. pseudograminearum* alone and the combination of strain YB-15 and *F. pseudograminearum*. In addition, the genome of *B. subtilis* YB-15 was sequenced and assembled by Nanopore and Illumina sequencing, resulting in a complete circular genome of 4,233,040 bp with GC content of 43.52% consisting of 4207 protein-encoding genes. Sequencing the genome of strain YB-15 was used to conduct an average nucleotide identity (ANI) analysis to determine the species as well as identify a number of genes for traits commonly associated with PGPB and BCA.

## 2. Materials and Methods

### 2.1. Bacteria and Pathogens Used in this Study

Five rhizospheric soil samples were collected from a commercial wheat field in Lushan, Henan Province, China, and strain YB-15 was isolated from wheat rhizospheric soil attached to roots at 5 cm beneath the soil surface as previously described [24]. An isolate of *F. pseudograminearum*, WZ001, was obtained from the Institute of Plant Protection Research, Henan Academy of Agricultural Sciences, Zhengzhou, China.

### 2.2. Detection of In Vitro Plant Growth Promotion and Biocontrol Traits

IAA production was detected with strain YB-15 colonies grown in L-tryptophan nutrient broth [25]. Colonies of strain YB-15 were grown on Chrome Azurol S blue agar to determine siderophore production [26], phosphorus agar to determine phosphorus solubilization [27], β-glucan agar to determine β-glucanaseactivity [28], starch agar to determine amylase activity [29], skim milk agar to determine protease activity [30] and carboxymethylcellulose agar to determine cellulase activity [28].

### 2.3. Growth Promotion and Biocontrol Assay of Strain YB-15 against Fusarium Crown Rot of Wheat Seedlings

*F. pseudograminearum* isolate WZ001 was grown on PDA for 5 days at 28 °C, and then six 5 mm agar plugs were transferred from the growing edge to a 1000 mL flask with 200 g sterilized boiled wheat grain and sand (3:1, *v*/*v*) and incubated at 28 °C for 7 days [31,32]. Strain YB-15 was grown in 100 mL LB broth with 180 rpm shaking at 37 °C until an OD595 nm of 0.8. Wheat cultivar zhengmai 366 seeds were surface-sterilized in 75% ethanol (*v*/*v*) for 30 s, rinsed three times with sterile distilled water and air-dried at room temperature [33]. The seeds were then soaked with strain YB-15 suspension for 12 h at 28 °C. A total of 25 seeds were sown per pot (10 cm diameter,10 cm high) containing 350 g mixture of sterile soil with or without 5% inoculum (*w*/*w*), and the pots were maintained in a greenhouse at 25 °C under a 12 h light/12 h dark photoperiod. The treatments were: (1) seeds soaked with YB-15 and planted in sterile soil without pathogen inoculum; (2) seeds not soaked with YB-15 and planted in sterile soil without pathogen inoculum; (3) seeds soaked with YB-15 and planted in sterile soil with pathogen inoculum; and (4) seeds not soaked with YB-15 and planted in sterile soil with pathogen inoculum. The *Fusarium* crown rot disease severity was assessed using scale of 0–4 at 20 days post-planting, and disease index (DI) was calculated using DI = [(0 × S0) + (1 × S1) + (2 × S2) + (3 × S3)]/A, where S is the number of wheat seedlings for each disease class and A is total number of tested wheat seedlings [34]. Control efficacy (CE) was calculated using CE= [(DI of control−DI of treatment)/DI of control] ×100% [24]. Each treatment had 20 wheat seedlings with six replicates. At 20 days post-planting, shoot height and root length were determined manually with a ruler, and root and shoot fresh weights were weighted with an electronic analytical balance (ME203E, Mettler Toledo, Changzhou, China).

### 2.4. Wheat Defense-Related Enzyme Activities

After 20 days post-planting, wheat leaves were harvested into liquid nitrogen and stored at −80 °C. Firstly, 1 g leaves of wheat seedlings were ground in pre-chilled mortar, and then transferred into a new 1.5 mL Eppendorf tube with 1 mL extraction buffer. After centrifugation for 10 min at 8000× *g*, the supernatant was transferred to another new 1.5 mL Eppendorf tube for detecting enzyme activities. Enzyme activities were measured by using commercially available assay kits of LOX (Cat. No. BC0325), PAL (Cat. No. BC0215), CAT (Cat. No. BC0205), PPO (Cat. No. BC0195) and POD (Cat. No. BC0095) accordingto the manufacturer’s instructions (Solarbio, Beijing, China). Absorbance was recorded with a plate reader (Tecan Spark, Tecan, Männedorf, Switzerland).

### 2.5. DNA Preparation, Genome Sequencing, Assembly and Annotation of Strain YB-15

Strain YB-15 was grown in NB medium on a rotary shaker (QYC-200, Fuma, Shanghai, China) with shaking speed of 180 rpm at 28 °C for 18 h. Genomic DNA of strain YB-15 was extracted by using the Mini-BEST Bacterial Genomic DNA Extraction Kit Ver. 3.0 (Takara, Beijing, China) according to the manufacturer’s directions. Two separate genomic DNA libraries were constructed for the Oxford Nanopore and Illumina NovaSeq sequencing systems. Genome assembly was conducted by Unicycler v0.4.9 [35]. A circular map of the strain YB-15 genome was generated by CGView server [36]. Nanopore and Illumina sequencing reads were mapped to the genome using Minimap2 (2.17-r974-dirty) [37] and BWA (0.7.17-r1198-dirty) [38], respectively. The depth of genome coverage was estimated with SAMtools [39]. Annotation of strain YB-15 genome was performed with Prokka (1.13) [40], and protein-coding, tRNA and rRNA genes were predicted using Prodigal (v2.6.3) [41], Aragorn (v1.2.38) [42] and RNAmmer (v1.2) [43], respectively.

### 2.6. Molecular Identification of Strain YB-15

The genome sequences of *Bacillus velezensis* FZB42, *Bacillus subtilis* H1, *Bacillus velezensis* FJAT-45028, *Bacillus subtilis* 168, *Bacillus licheniformis* ATCC 14580, *Bacillus altitudinis* GQYP101, *Bacillus licheniformis* SRCM103583, *Bacillus altitudinis* CHB19, *Bacillus pumilus* ZB201701 and *Bacillus pumilus* SF-4 were obtained from the NCBI genome database (GenBank Accession Nos: CP000560.2, CP026662.1, CP047157.1, NC_000964.3, CP034569.1, CP040514.1, CP035404.1, CP043559.1, CP029464.1 and CP047089.1, respectively). ANI among the above genomes was analyzed with ANI calculator [44].

### 2.7. Analysis of CAZymes and Genes Associated with Growth Promotion and Secondary Metabolites of Strain YB-15

Annotated protein-coding sequences of strain YB-15 were aligned against the carbohydrate-active enzyme (CAZy) database using dbCAN2 with the threshold of *E*-value1e-15 [45]. Signal peptide was predicted by SignalP (v4.1) [46]. Gene clusters for synthesis of secondary metabolite were identified by antiSMASH [47]. Local BLASTP was used to identify genes associated with plant growth promotion.

### 2.8. Statistical Analysis

Statistical analysis was performed using SPSS v21.0 software according to the one-way analysis of variance. The differences among means were determined usingDuncan’s multiple range tests with *p* value ≤ 0.05.

## 3. Results

### 3.1. Isolation of Strain YB-15 and Its Antagonism against F. pseudograminearum

**Figure 1:**
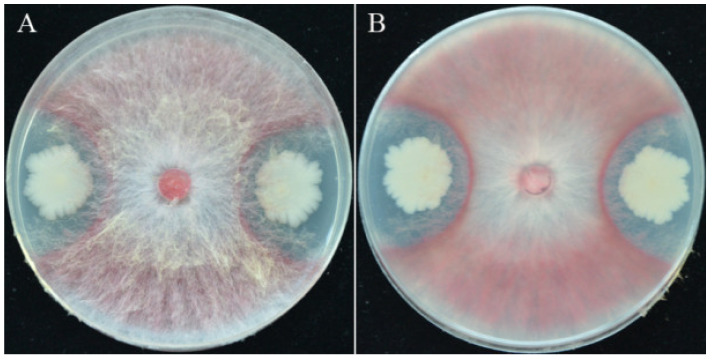
Characteristics of strain YB-15: (**A**,**B**) Dual culture of *F. pseudograminearum* and strain YB-15 on PDA.

A number of 78 bacterial strains were isolated and screened from wheat rhizospheric soil by dilution plating. Out of the 78 strains, 59 showed varying levels of antagonism against *F. pseudograminearum* in dual PDA culture (data not shown). Strain YB-15 was selected for further investigation because it showed the greatest antagonism against *F. pseudograminearum* on PDA (Figure 1A,B). Strain YB-15 colonies were opaque white with rod-shaped and Gram-positive (data not shown).

### 3.2. Plant Growth Promotion and Antifungal Traits of Strain YB-15 in Culture

**Figure 2:**
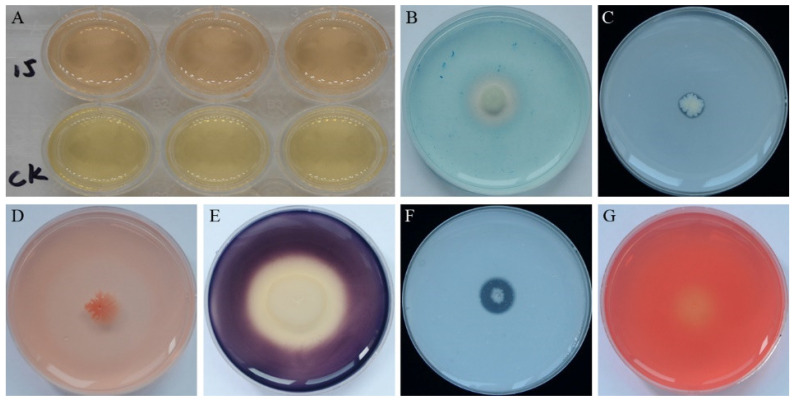
PGP and Antifungal traits of strain YB-15: (**A**) The upper indicating IAA production by pink coloration; (**B**) Yellow-orange halos indicating siderophore production; (**C**) Phosphorus solubilization indicated by a halo zone around strain YB-15 colonies; (**D**) β-1, 3-glucanase activity indicated by a zone around strain YB-15 colonies; (**E**) Amylase activity indicated by a clear zone around strain YB-15 colonies; (**F**) Protease activity indicated by an obvious hydrolytic zone around strain YB-15 colonies; (**G**) Cellulase activity indicated by a zone of hydrolysis around strain YB-15 colonies.

Possible plant growth promotion traits of strain YB-15 detected in culture were the production of indole acetic acid (IAA) (Figure 2A), siderophore (Figure 2B) and phosphorus solubilization (Figure 2C). Potential antifungal traits detected in culture were secretion of β-1, 3-glucanase (Figure 2D), amylase (Figure 2E), protease (Figure 2F) and cellulase (Figure 2G).

### 3.3. Effects of Strain YB-15 on Fusarium Crown Rot of Wheat Seedlings

**Figure 3:**
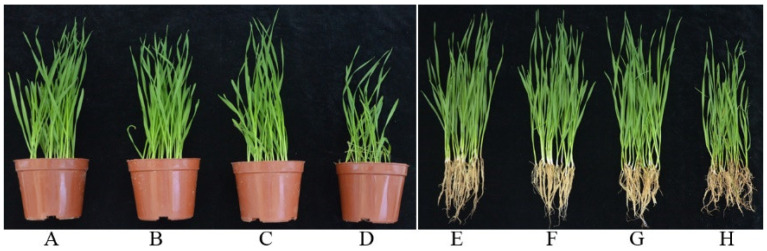
Effect of strain YB-15 against *Fusarium* crown rot caused by *F.pseudograminearum* and on growth of wheat seedlings: (**A**,**E**) seeds soaked with YB-15 and planted in sterile soil without pathogen inoculum; (**B**,**F**) seeds not soaked with YB-15 and planted in sterile soil without pathogen inoculum; (**C**,**G**) seeds soaked with YB-15 and planted in sterile soil with pathogen inoculum; (**D**,**H**) seeds not soaked with YB-15 and planted in sterile soil with pathogen inoculum.

Typical *Fusarium* crown rot symptoms with brown lesions on the stems were observed in *F. pseudograminearum* inoculated seedlings at 20 days post-planting, but no disease symptoms were observed in seedlings with both *F. pseudograminearum* and strain YB-15 or control treatments not inoculated with *F. pseudograminearum* (Figure 3). The disease severity of wheat seedlings infected with *F. pseudograminearum* was 2.63 ± 0.03, whereas it was only 0.48 ± 0.02 with the combination of strain YB-15 and *F. pseudograminearum*. Disease incidence was 91.67 ± 1.67% infected with *F. pseudograminearum*, but only 15.00 ± 2.89% with the combination of strain YB-15 and *F. pseudograminearum*. Control efficacy was 81.50 ± 0.76% with seed treatment of strain YB-15 compared to non-treated control seeds infected with *F. pseudograminearum* (Table 1).

### 3.4. Growth Promotion of Wheat Seedlings by Strain YB-15

After 20 days post-planting, the shoot and root fresh weight of seedlings treated with strain YB-15 was significantly increased by 4.2% and 11.4%, respectively; although, root length and shoot height were not significantly affected (Table 2). Inoculation with *F. pseudograminearum* resulted in significantly lower shoot height and weight as well as root length and weight, compared to the control. Most notable was the reduction in root fresh weight by 46.7%. Inoculation with both strain YB-15 and *F. pseudograminearum* resulted in significantly higher shoot height and weight as well as root weight and length, compared to that of the *F. pseudograminearum* inoculated wheat seedlings. There was no significant difference in shoot height and root length between the non-treated control and seedlingsinoculated with both strain YB-15 and *F. pseudograminearum*; although, the fresh weights remained lower.

### 3.5. Defense-Related Enzyme Activities of Wheat Seedlings

Wheat seedlings with strain YB-15 treatment exhibited significantly higher activities of PAL, POD, CAT and PPO, but not LOX, compared to non-treated control seedlings (Table 3). Inoculation with *F. pseudograminearum* resulted in significantly higher PAL, POD, CAT, PPO and LOX activities, compared to non-treated seedlings, but the enzyme activities were significantly higher with the combination of strain YB-15 and *F. pseudograminearum* compared to only *F. pseudograminearum* inoculation. The activities with the combination of strain YB-15 and *F. pseudograminearum* were also significantly higher compared to strain YB-15 alone, except for POD activity, which was significantly lower.

### 3.6. Genome Assembly and Annotation of Strain YB-15

**Figure 4:**
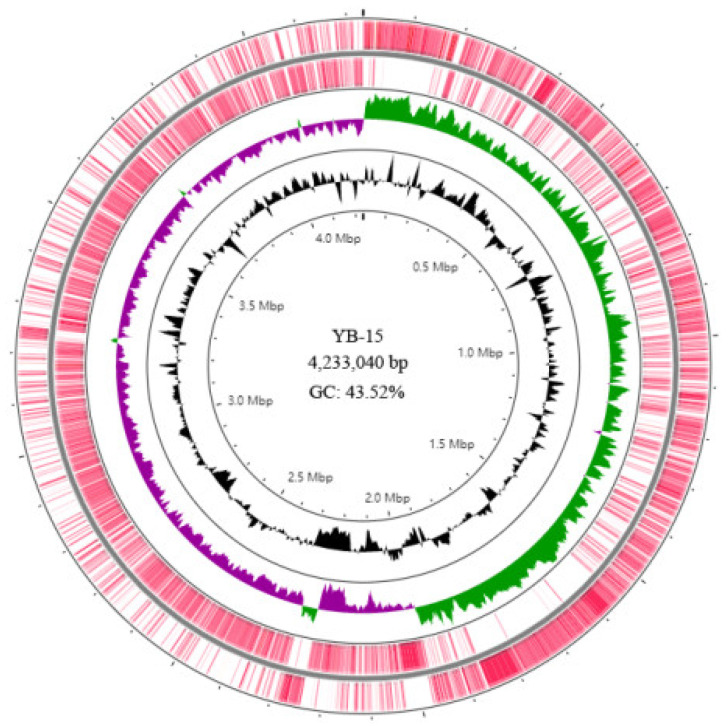
Circular map of *B. subtilis* YB-15 genome visualized by CGView Server. The distribution of rings from outwards to inwards be: ring 1 and 2 for protein-coding genes on the forward strand and reverse strand; ring 3 for GC skew in plus (green) and GC skew in minus (purple); ring 4 for GC content (black).

A total of 21,392 long reads containing 1,000,070,603 bases with a mean length of 46,749.7 bp and an N50 of 45,733 bp were obtained by Nanopore sequencing, and 13,740,276 reads containing 2,061,041,400 bases were generated by Illumina sequencing. These were used to assemble the genome of strain YB-15 with 238.54X and 486.61X genome coverage for the Nanopore and Illumina sequencing data, respectively. The completed assembled genome of strain YB-15 was deposited at GenBank with Accession number CP092631. The strain YB-15 genome was a 4,233,040 bp single circular chromosome with 43.52% GC content (Figure 4). There were 4207 protein-encoding genes, 86 tRNAs and 27 rRNAs, which were annotated (Table S1).

### 3.7. Species Identification of Strain YB-15

**Figure 5:**
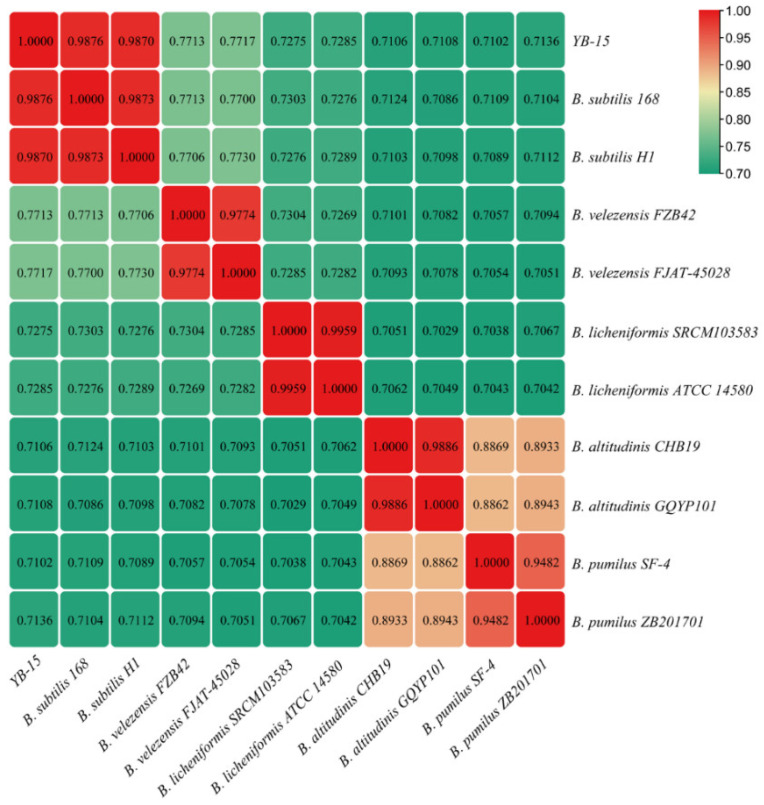
Heatmap of ANIvalues based on whole-genome sequences of *B. subtilis* YB-15 and 10 other *Bacillus* species.

ANI values of the genome of strain YB-15 to those of ten other *Bacillus* strains ranged from 71.02 to 98.76% (Figure 5). The ANI values between strain YB-15 and *Bacillus subtilis* strain 168 was 98.76% and between strain YB-15 and *Bacillus subtilis* strain H1 was 98.70%, both of which were above the thresholds of 95–96% for recognizing prokaryotic species boundaries [48]. Thus, strain YB-15 was identified as *Bacillus subtilis*.

### 3.8. Predicted Genes for CAZymes

The *B. subtilis* YB-15 genome had 122 putative genes encoding CAZymes, including 48 glycoside hydrolases (GHs), 19 carbohydrate esterases (CEs), 7 polysaccharide lyases (PLs), 43 glycosyltransferases (GTs), 7 carbohydrate-binding modules (CBMs) and 4 auxiliary activities (AAs) (Table S2). Among those, six genes are classified to be both GHs and CBMs. There were 28 genes predicted to have signal peptides, and most belonged to the GHs (17) followed by CBMs (5), PLs (5), CEs (4) and GTs (1) with 4 of them having both GHs and CBMs.

### 3.9. Predicted Genes for Secondary Metabolites

The *B. subtilis* YB-15 genome had 11 putative gene clusters for secondary metabolites (Table 4). For antimicrobial compound synthesis, there were five predicted gene clusters with 100% similarity to clusters for the synthesis of bacillibactin, fengycin, bacillaene, subtilosin A and bacilysin. There was also one gene cluster with 82% similarity to the gene cluster for surfactin synthesis and one gene cluster with 60% similarity to the gene cluster for paenibacterin synthesis. Four other predicted gene clusters were only identified to type with no highly similar known clusters in the antiSMASH database. Two were for terpenes, one was for a type III polyketide synthase, and one was for atRNA-dependent cyclodipeptide synthase.

### 3.10. Predicted Genes for Plant Growth Promotion

In the *B. subtilis* YB-15 genome, there were genes possibly involved in plant growth promotion related to nutrient acquisition, phytohormone production, chemotaxis and motility (Table S3). Genes associated with nutrient acquisition were all the genes in the *nasABCDEF* operon for nitrate/nitrite assimilation, *KtrAB* and *KtrCD* for potassium uptake, *phoP* and *phoR* in the *phoPR* operon as well as *phoB*, *phoA* and *phoD* for phosphate assimilation and all the genes in the *PstABCS* operon for an ABC-type phosphate transport system (Table S3). Genes associated with phytohormone production were all the genes in the *trpABCDEF* operon as well as *yclC* for IAA biosynthesis; *miaA* and *miaB* for cytokinin biosynthesis; *speA* and *speB* for putrescine biosynthesis; *speD* and *speE* for spermidine biosynthesis; and *alsS*, *alsD*, *ilvB*, *ilvH* and *bdhA* for acetoin and butanediol biosynthesis. For chemotaxis, there were *cheA, cheB, cheD, cheR, cheY,* and *cheW* genes related to the two-component sensor kinase, MCP-glutamate methylesterase, protein deaminase, chemotaxis protein methyltransferase, two-component response regulator and CheA modulator chemotaxis, respectively. For motility, there were genes associated with biosynthesis and regulation of flagellum assembly. These were *flgD*, *flgE*(*flgG*), *flgK*, *flgL*, *hag* (*fliC*), *fliD* for the hook and filament; *fliH*, *fliP*, *fliI*, *fliR*, *fliQ*, *flhB* and *flhA* for flagellar protein secretion; *flgB*, *flgC* and *fliE* for the proximal rod; *fliF*, *fliG*, *fliM* and *fliN(fliY)* for the MS and C ring; *flgM*, *flgN*, *fliK*, *fliJ*, *flit* and *fliS* for other flagellar proteins; and *motA* and *motB* for the rotary motor.

## 4. Discussion

In this study, *B. subtilis* YB-15, isolated from wheat rhizospheric soil, significantly reduced *Fusarium* crown rotcaused by *F. pseudograminearum* with a control efficacy of 81.50%. *B. subtilis* YB-15 also increased shoot fresh weight by 13.7% and root fresh weight by 70.3% relative to *F. pseudograminearum* inoculated wheat seedlings. This was similar to the control efficacy of 80.33% for *F. pseudograminearum* crown and root rot of wheat seeds with *B. subtilis* strain UTBMS7 inoculation, which also significantly increased wheat stem length and root dry weight by 68 and 64%, respectively, compared to *F. pseudograminearum* inoculated wheat seedlings [19]. For *F. pseudograminearum* crown rot of sorghum, *Bacillus velezensis* N54 significantly decreased crown rot incidence by 55.6%, which was the most among the rhizobacterial isolates tested. However, it did not improve plant growth as infected plants and strain N54 had significantly lowers hoot and root weight by 14 and 35%, respectively, compared to plants infected with only *F. pseudograminearum* [20].

In addition, *B. subtilis* YB-15 also promoted plant growth in the absence of infection by *F. pseudograminearum*. Shoot fresh weight was increased by 4.2% and root fresh weight increased by 11.4% at 20 days in this study, which was less than the increase in wheat with *B. subtilis* strain UTBMS7, where the strain significantly increased wheat stem and root dry weight by 117 and 107%, respectively, without *F. pseudograminearum* [19]. However, neither this study nor that of Sasani and Ahmadzadeh (2021) showed a significant increase in stem height or root length. For other studies of *Bacillus* species in promoting wheat growth, Chanway et al. (1988) showed a significant increase in root dry weight of 37.8% and shoot height of 2.8% but not shoot dry weight in wheat cv.Katepwa with *Bacillus* strain 5A1; Akinrinlola et al., (2018) showed a significant increase in shoot height of 36.5% but no significant increase in root and shoot fresh weights with *Bacillus megaterium* strain R181; Ku et al., (2018) showed a significant increase in shoot fresh weight of 77% and root fresh weight of 177% with *Bacillus cereus* strain YL6; and Rojas Padilla et al., (2020) showed a significant increase of 27% in shoot dry weight and 30% in root dry weight with *B. megaterium* strain TRQ8. Thus, there is considerable variation among *Bacillus* strains for their ability to improve wheat growth, which could be due to differences in the strains as well as in the types of wheat used in each study as Chanway et al., (1988) showed a plant genotypic effect on plant growth promotion with *Bacillus* isolates being able to enhance root growth of cv. Katepwa but having no effect on root growth of the parental cv. Neepawa.

The biocontrol potential of *B. subtilis* against plant pathogens could be due to its direct effects on a pathogen, such as by producing antimicrobial compounds and hydrolytic enzymes, or indirect effects on the pathogen by inducing host systemic resistance [12,49,50,51]. For antimicrobial compounds, *B. subtilis* YB-15 has genes to produce the antimicrobial compounds bacillaene, fengycin, bacillibactin, surfactin, subtilosinA, bacilysin and paenibacterin. For biocontrol agents of *Fusarium* species, there are many examples of them having genes for the production of such compounds, such as *B. velezensis* LM2303 for the cyclic lipopeptides fengycin and surfactin [52], and *B. subtilis* SEM-2 for fengycin and surfactin as well as the polyene bacillaene, catechol-based siderophore bacillibactin, cyclic peptide subtilosin A, and dipeptide bacilysin [53]. A gene cluster also with 60% similarity to paenibacterin biosynthesis was found in *Paenibacillus* *polymyxa* WLY78, which was a biocontrol agent of *Fusarium* wilt of cucumber [54]. Although found in several *Paenibacillus* species, this appears to be the first report of this type of lipopeptide possibly produced by a *Bacillus* species. For hydrolytic enzymes, *B. subtilis* YB-15 was able to secrete β-1, 3-glucanase, amylase, protease and cellulase, which are able to degrade various cell wall components of fungal pathogens [55]. Furthermore, 122 putative genes encoding CAZymes were found in the *B. subtilis* YB-15 genome, some of which could act against fungal pathogens [12]. *Bacillus* species are well-known inducers of systemic resistance in plants [56,57], and *B. subtilis* YB-15 also appears to be able to induce systemic host resistance as indicated by seed inoculation resulting in significant increases in the activities of several defense enzymes in leaves of wheat seedlings, which may contribute to the control of *Fusarium* crown rot in this study.

For the promotion of plant growth, *Bacillus subtilis* possesses several mechanisms, such as the production of phytohormones and siderophores, increasing tolerance to abiotic stresses and improved nutrient acquisition [11,12,15,50]. Production of IAA by *B. subtilis* YB-15 could increase cell elongation and production of cytokinin could increase cell division, both of which result in increased size of plant roots and shoots [11]. Improved plant growth and root development could be due to the production of the polyamines putrescine and spermidine that can act by regulating expansin and ethylene levels, and the production of the related C4 compounds acetoin and butanediol that can act through altering auxin and cytokinin homeostasis [12]. Production of siderophores by *B. subtilis* YB-15 can chelate iron thus promoting plant growth by making iron more available [58]. Other genes in the *B. subtilis* YB-15 genome that can directly promote growth through enhanced nutrient availability were for nitrate/nitrite assimilation, potassium uptake and phosphate assimilation and transport [11]. In addition, genes for flagellar motility and chemotaxis in the *B. subtilis* YB-15 genome indicate that they can likely identify root exudates and migrate to the roots, which is common among effective PGPB and BCA [12].

## 5. Conclusions

In summary, *B. subtilis* YB-15 is a promising BCA exhibiting significant biocontrol effects against *Fusarium* crown rot caused by *F. pseudograminearum*, which could be due to the production of hydrolytic enzymes, antimicrobial compounds and inducing host systemic resistance. It is also a promising PGPB demonstrating growth promotion of wheat seedlings both with and without *F. pseudograminearum* infection, which could be due to the potential of strain YB-15 to produce phytohormone and siderophores and improve nutrient acquisition for wheat seedlings. While in vitro and in planta tests can reveal common mechanisms employed by BCA and PGPB, genome sequencing can rapidly determine a much wider range of molecular mechanisms underlying BCA and PGPB-mediated biocontrol and growth promotion.

## Figures and Tables

**Table 1:** Disease index, disease incidence and control efficacy of *B. subtilis* YB-15 against *Fusarium* crown rot of wheat.

Treatments	Disease Incidence (%)	Disease Index	Control Efficacy (%)
FPS	91.67 ± 1.67 a	2.63 ± 0.03 a	
FPS + YB-15	15.00 ± 2.89 b	0.48 ± 0.02 b	81.50 ± 0.76

Note: Data in the table are mean ± standard deviation; the column distribution of different letters was significantly different with the level of *p* < 0.05.

**Table 2:** Effects of *B. subtilis* YB-15 on the growth of wheat seedlings.

Treatments	Shoot Height (cm)	Root Length (cm)	Root Fresh Weight (g)	Shoot Fresh Weight (g)
CK	27.98 ± 0.76 a	8.74 ± 1.11 a	1.05 ± 0.02 b	5.32 ± 0.06 b
YB-15	28.33 ± 0.77 a	8.87 ± 0.97 a	1.17 ± 0.03 a	5.54 ± 0.03 a
FPS	25.52 ± 2.73 b	7.1 ± 0.98 b	0.56 ± 0.03 d	4.01 ± 0.03 d
FPS + YB-15	27.7 ± 0.79 a	8.96 ± 1.39 a	0.94 ± 0.02 c	4.56 ± 0.04 c

Note: Data in the table are mean ± standard deviation; the column distribution of different letters (a–d) was significantly different with the level of *p* < 0.05.

**Table 3:** Activities of defense-related enzymes in wheat leaves.

Treatments	LOX (U/g)	PAL (U/g)	CAT (U/g)	PPO (U/g)	POD (U/g)
CK	574.76 ± 4.48 c	28.57 ± 0.54 d	213.07 ± 1.39 d	22.25 ± 0.87 d	8173.10 ± 47.64 d
YB-15	733.42 ± 7.04 c	32.30 ± 0.74 c	274.24 ± 0.90 c	32.98 ± 0.90 c	14,955.53 ± 131.38 b
FPS	6581.31 ± 72.21 b	34.64 ± 0.55 b	373.10 ± 1.21 b	41.61 ± 0.82 b	8657.33 ± 47.98 c
FPS + YB-15	7457.08 ± 69.68 a	41.61 ± 0.60 a	597.35 ± 0.96 a	49.71 ± 0.89 a	12,425.40 ± 172.51 a

Note: Data in the table are mean ± standard deviation; the column distribution of different letters (a–d) demonstrated significant difference at *p* values < 0.05 level.

**Table 4:** The putative gene clusters for synthesis of secondary metabolites in *B. subtilis* YB-15 genome analyzed by antiSMASH.

Clusters	Types	From	To	Most Similar Known Clusters	Similarity
Cluster 1	NRPS	359,999	423,558	surfactin	82%
Cluster 2	terpene	1,127,798	1,148,314		
Cluster 3	NRPS	1,557,678	1,624,493	paenibacterin	60%
Cluster 4	NRPS	1,770,137	1,875,386	bacillaene	100%
Cluster 5	NRPS, betalactone	1,969,943	2,047,024	fengycin	100%
Cluster 6	terpene	2,119,954	2,141,852		
Cluster 7	T3PKS	2,318,835	2,359,932		
Cluster 8	NRPS	3,262,252	3,309,388	bacillibactin	100%
Cluster 9	CDPS	3,592,205	3,612,951		
Cluster 10	sactipeptide	3,842,693	3,864,304	subtilosin A	100%
Cluster 11	other	3,871,089	3,912,507	bacilysin	100%

## Data Availability

The completed assembled genome of strain YB-15 was deposited at GenBank with Accession No. CP092631.

## Supplementary Materials

The following supporting information can be downloaded at: https://www.mdpi.com/article/10.3390/biology11050778/s1, Table S1: Genome annotation of *B. subtilis* YB-15; Table S2: CAZymes identified in the *B. subtilis* YB-15 genome. Table S3: Genes associated with plant growth promotion identified in the strain YB-15 genome.

## References

[1] Alomari D.Z., Eggert K., von Wiren N., Alqudah A.M., Polley A., Plieske J., Ganal M.W., Pillen K., Roder M.S. (2018). Identifying Candidate Genes for Enhancing Grain Zn Concentration in Wheat. Front. Plant Sci..

[2] Moya E. (2013). Fusarium crown rot disease: Biology, interactions, management and function as a possible sensor of global climate change. Cienc. E Investig. Agrar. Rev. Latinoam. Cienc. Agric..

[3] Kazan K., Gardiner D.M. (2018). *Fusarium* crown rot caused by *Fusarium pseudograminearum* in cereal crops: Recent progress and future prospects. Mol. Plant Pathol..

[4] Nicolopoulou-Stamati P., Maipas S., Kotampasi C., Stamatis P., Hens L. (2016). Chemical Pesticides and Human Health: The Urgent Need for a New Concept in Agriculture. Front. Public Health.

[5] Sardar M.F., Abbas T., Naveed M., Siddique S., Mustafa A., Abbasi B., Khan K. (2021). Biopesticides: Importance and Challenges.

[6] Dehghanpour-Farashah S., Taheri P., Falahati-Rastegar M. (2019). Effect of polyamines and nitric oxide in *Piriformospora* indica-induced resistance and basal immunity of wheat against *Fusarium pseudograminearum*. Biol. Control..

[7] Kim Y.T., Monkhung S., Lee Y.S., Kim K.Y. (2019). Effects of *Lysobacter antibioticus* HS124, an effective biocontrol agent against *Fusarium graminearum*, on crown rot disease and growth promotion of wheat. Can. J. Microbiol..

[8] Winter M., Samuels P.L., Otto-Hanson L.K., Dill-Macky R., Kinkel L.L. (2019). Biological control of *Fusarium* crown and root rot of wheat by *Streptomyces* isolates—It’s complicated. Phytobiomes J..

[9] O’Sullivan C.A., Roper M.M., Myers C.A., Thatcher L.F. (2021). Developing Actinobacterial Endophytes as Biocontrol Products for *Fusarium pseudograminearum* in Wheat. Front. Bioeng. Biotechnol..

[10] Kumar P., Dubey R.C., Maheshwari D.K. (2012). *Bacillus* strains isolated from rhizosphere showed plant growth promoting and antagonistic activity against phytopathogens. Microbiol. Res..

[11] Miljakovic D., Marinkovic J., Balesevic-Tubic S. (2020). The Significance of *Bacillus* spp. in Disease Suppression and Growth Promotion of Field and Vegetable Crops. Microorganisms.

[12] Blake C., Christensen M.N., Kovacs A.T. (2021). Molecular Aspects of Plant Growth Promotion and Protection by *Bacillus subtilis*. Mol. Plant Microbe Interact..

[13] Ali M.A., Naveed M., Mustafa A., Abbas A., Kumar V., Kumar M., Sharma S., Prasad R. (2017). The Good, the Bad, and the Ugly of Rhizosphere Microbiome. Probiotics and Plant Health.

[14] Xu W., Zhang L., Goodwin P.H., Xia M., Zhang J., Wang Q., Liang J., Sun R., Wu C., Yang L. (2020). Isolation, Identification, and Complete Genome Assembly of an Endophytic *Bacillus velezensis* YB-130, Potential Biocontrol Agent Against *Fusarium graminearum*. Front. Microbiol..

[15] Saeed Q., Xiukang W., Haider F.U., Kučerik J., Mumtaz M.Z., Holatko J., Naseem M., Kintl A., Ejaz M., Naveed M. (2021). Rhizosphere Bacteria in Plant Growth Promotion, Biocontrol, and Bioremediation of Contaminated Sites: A Comprehensive Review of Effects and Mechanisms. Int. J. Mol. Sci..

[16] Chanway C.P., Nelson L.M., Holl F.B. (1988). Cultivar-specific growth promotion of spring wheat (*Triticum aestivum* L.) by coexistent *Bacillus* species. Can. J. Microbiol..

[17] Ku Y., Xu G., Tian X., Xie H., Yang X., Cao C., Chen Y. (2018). Root colonization and growth promotion of soybean, wheat and Chinese cabbage by *Bacillus cereus* YL6. PLoS ONE.

[18] Rojas Padilla J., Chaparro Encinas L.A., Robles Montoya R.I., de los Santos Villalobos S. (2020). Growth promotion on wheat (*Triticum turgidum* L. subsp. *durum*) by co-inoculation of native *Bacillus* strains isolated from the Yaqui Valley, Mexico. Nova Scientia.

[19] Sasani m., Ahmadzadeh M. (2021). Evaluation of antagonistic effect of several strain of *Bacillus* bacteria on control of crown and root rot of wheat with *Fusarium pseudograminearum*. Biol. Control Pests Plant Dis..

[20] Carlson R., Tugizimana F., Steenkamp P.A., Dubery I.A., Hassen A.I., Labuschagne N. (2020). Rhizobacteria-induced systemic resilience in *Sorghum bicolor* (L.) moench against *Fusarium pseudograminearum* crown rot under drought stress conditions. Biol. Control.

[21] Wu J.J., Chou H.P., Huang J.W., Deng W.L. (2021). Genomic and biochemical characterization of antifungal compounds produced by *Bacillus subtilis* PMB102 against *Alternaria brassicicola*. Microbiol. Res..

[22] Kiesewalter H.T., Lozano-Andrade C.N., Wibowo M., Strube M.L., Maroti G., Snyder D., Jorgensen T.S., Larsen T.O., Cooper V.S., Weber T. (2021). Genomic and Chemical Diversity of *Bacillus subtilis* Secondary Metabolites against Plant Pathogenic Fungi. mSystems.

[23] Franco-Sierra N.D., Posada L.F., Santa-Maria G., Romero-Tabarez M., Villegas-Escobar V., Alvarez J.C. (2020). *Bacillus subtilis* EA-CB0575 genome reveals clues for plant growth promotion and potential for sustainable agriculture. Funct. Integr. Genom..

[24] Yang L., Quan X., Xue B., Goodwin P.H., Lu S., Wang J., Du W., Wu C. (2015). Isolation and identification of *Bacillus subtilis* strain YB-05 and its antifungal substances showing antagonism against *Gaeumannomyces graminis* var. tritici. Biol. Control.

[25] Glickmann E., Dessaux Y. (1995). A critical examination of the specificity of the Salkowski reagent for indolic compounds produced by phytopathogenic bacteria. Appl. Environ. Microbiol..

[26] Schwyn B., Neilands J.B. (1987). Universal chemical assay for the detection and determination of siderophores. Anal. Biochem..

[27] Chen Q., Liu S. (2019). Identification and Characterization of the Phosphate-Solubilizing Bacterium *Pantoea* sp. S32 in Reclamation Soil in Shanxi, China. Front. Microbiol..

[28] Teather R.M., Wood P.J. (1982). Use of Congo red-polysaccharide interactions in enumeration and characterization of cellulolytic bacteria from the bovine rumen. Appl. Environ. Microbiol..

[29] Al-Naamani L.S.H., Dobretsov S., Al-Sabahi J., Soussi B. (2015). Identification and characterization of two amylase producing bacteria *Cellulosimicrobium* sp. and *Demequina* sp. isolated from marine organisms. J. Agric. Mar. Sci. [JAMS].

[30] Kazanas N. (1968). Proteolytic activity of microorganisms isolated from freshwater fish. Appl. Microbiol..

[31] Zhang J., Wang L.M., Li Y.H., Ding S.L., Yuan H.X., Riley I.T., Li H.L. (2016). Biocontrol of cereal cyst nematode by *Streptomyces anulatus* isolate S07. Australas. Plant Pathol..

[32] Xu W., Zhao F., Deng X., Goodwin P., Xia M., Zhang J., Sun R., Liang J., Wu C., Yang L. (2022). First Report of Collar Canker and Dieback of *Camellia sinensis* caused by *Fusarium solani* Species Complex in Henan, China. Plant Dis..

[33] Xu W., Xu L., Deng X., Goodwin P.H., Xia M., Zhang J., Wang Q., Sun R., Pan Y., Wu C. (2021). Biological Control of Take-All and Growth Promotion in Wheat by *Pseudomonas chlororaphis* YB-10. Pathogens.

[34] Ullah H., Yasmin H., Mumtaz S., Jabeen Z., Naz R., Nosheen A., Hassan M.N. (2020). Multitrait *Pseudomonas* spp. Isolated from Monocropped Wheat (*Triticum aestivum*) Suppress *Fusarium* Root and Crown Rot. Phytopathology.

[35] Wick R.R., Judd L.M., Gorrie C.L., Holt K.E. (2017). Unicycler: Resolving bacterial genome assemblies from short and long sequencing reads. PLoS Comput. Biol..

[36] Stothard P., Wishart D.S. (2004). Circular genome visualization and exploration using CGView. Bioinformatics.

[37] Li H. (2018). Minimap2: Pairwise alignment for nucleotide sequences. Bioinformatics.

[38] Li H. (2013). Aligning sequence reads, clone sequences and assembly contigs with BWA-MEM. arXiv.

[39] Li H., Handsaker B., Wysoker A., Fennell T., Ruan J., Homer N., Marth G., Abecasis G., Durbin R., 1000 Genome Project Data Processing Subgroup (2009). The Sequence Alignment/Map format and SAMtools. Bioinformatics.

[40] Seemann T. (2014). Prokka: Rapid prokaryotic genome annotation. Bioinformatics.

[41] Hyatt D., Chen G.L., Locascio P.F., Land M.L., Larimer F.W., Hauser L.J. (2010). Prodigal: Prokaryotic gene recognition and translation initiation site identification. BMC Bioinform..

[42] Laslett D., Canback B. (2004). ARAGORN, a program to detect tRNA genes and tmRNA genes in nucleotide sequences. Nucleic Acids Res..

[43] Lagesen K., Hallin P., Rodland E.A., Staerfeldt H.H., Rognes T., Ussery D.W. (2007). RNAmmer: Consistent and rapid annotation of ribosomal RNA genes. Nucleic Acids Res..

[44] Yoon S.H., Ha S.M., Lim J., Kwon S., Chun J. (2017). A large-scale evaluation of algorithms to calculate average nucleotide identity. Antonie Van Leeuwenhoek.

[45] Zhang H., Yohe T., Huang L., Entwistle S., Wu P., Yang Z., Busk P.K., Xu Y., Yin Y. (2018). dbCAN2: A meta server for automated carbohydrate-active enzyme annotation. Nucleic Acids Res..

[46] Petersen T.N., Brunak S., von Heijne G., Nielsen H. (2011). SignalP 4.0: Discriminating signal peptides from transmembrane regions. Nat. Methods.

[47] Blin K., Shaw S., Steinke K., Villebro R., Ziemert N., Lee S.Y., Medema M.H., Weber T. (2019). antiSMASH 5.0: Updates to the secondary metabolite genome mining pipeline. Nucleic Acids Res..

[48] Richter M., Rossello-Mora R. (2009). Shifting the genomic gold standard for the prokaryotic species definition. Proc. Natl. Acad. Sci. USA.

[49] Caulier S., Nannan C., Gillis A., Licciardi F., Bragard C., Mahillon J. (2019). Overview of the Antimicrobial Compounds Produced by Members of the *Bacillus subtilis* Group. Front. Microbiol..

[50] Khan N., Ali S., Shahid M.A., Mustafa A., Sayyed R.Z., Curá J.A. (2021). Insights into the Interactions among Roots, Rhizosphere, and Rhizobacteria for Improving Plant Growth and Tolerance to Abiotic Stresses: A Review. Cells.

[51] Salwan R., Sharma V. (2020). Genome wide underpinning of antagonistic and plant beneficial attributes of *Bacillus* sp. SBA12. Genomics.

[52] Chen L., Heng J., Qin S., Bian K. (2018). A comprehensive understanding of the biocontrol potential of *Bacillus velezensis* LM2303 against Fusarium head blight. PLoS ONE.

[53] Li Q., Liao S., Wei J., Xing D., Xiao Y., Yang Q. (2020). Isolation of *Bacillus subtilis* strain SEM-2 from silkworm excrement and characterisation of its antagonistic effect against *Fusarium* spp.. Can. J. Microbiol..

[54] Li Y., Chen S. (2019). Fusaricidin Produced by *Paenibacillus polymyxa* WLY78 Induces Systemic Resistance against *Fusarium* Wilt of Cucumber. Int. J. Mol. Sci..

[55] Jadhav H., Sayyed R. (2016). Hydrolytic enzymes of rhizospheric microbes in crop protection. MOJ Cell Sci. Rep..

[56] Choudhary D.K., Johri B.N. (2009). Interactions of *Bacillus* spp. and plants—With special reference to induced systemic resistance (ISR). Microbiol. Res..

[57] Kloepper J.W., Ryu C.M., Zhang S. (2004). Induced Systemic Resistance and Promotion of Plant Growth by *Bacillus* spp.. Phytopathology.

[58] Scavino A.F., Pedraza R.O. (2013). The role of siderophores in plant growth-promoting bacteria. Bacteria in Agrobiology: Crop Productivity.

